# Impact of the COVID-19 Pandemic on Consumers’ Access to Essential Medicines in Nigeria

**DOI:** 10.4269/ajtmh.20-0838

**Published:** 2020-08-18

**Authors:** Nwoke Emmanuel Awucha, Ofomata Chinelo Janefrances, Amadi Chima Meshach, Jibuaku Chiamaka Henrietta, Akahome Ibilolia Daniel, Nwagbo Esther Chidiebere

**Affiliations:** 1 Faculty of Pharmaceutical Sciences, University of Port Harcourt, Port Harcourt, Nigeria;; 2 Faculty of Pharmaceutical Sciences, Nnamdi Azikiwe University, Awka, Nigeria;; 3 National Institute of Pharmaceutical Research and Development (NIPRD), Abuja, Nigeria;; 4 The Nigerian Police Medical Services, Port Harcourt, Nigeria;; 5 Medical Services, Nnamdi Azikiwe University, Awka, Nigeria

## Abstract

COVID-19 is a global pandemic which has seriously impacted the economy of nations. Access to essential medicines is of utmost importance. This study examined the impacts of the COVID-19 pandemic on the ease of access to essential medicines by end users. A cross-sectional survey using electronic questionnaires was conducted on study participants across the 36 states of Nigeria. They were assessed on sociodemographics, health characteristics, and challenges in accessing essential medicines during the COVID-19 pandemic. Data obtained were analyzed using the Statistical Package for the Social Sciences (SPSS version 20, IBM, Armonk, NY) with overall impact of the pandemic operationalized as < 60.0% or ≥ 60.0% access to essential medicines by respondents as maximal and minimal impact, respectively. The results showed that 35.2% of the respondents managing chronic illnesses had difficulties accessing essential medicines during the COVID-19 lockdown, with 84.0% experiencing deteriorating chronic health conditions in the light of difficulty in accessing their medicines. The proportion of respondents who sourced for orthodox medicines before COVID-19 lockdown (98.4%) was significantly (*P* < 0.05) higher than that of those who sourced for the same during the lockdown (89.0%). Increase in cost of medicines was observed by 77.7% of participants, with 73.9% of respondents living with chronic illness affirming that their income was negatively affected by the pandemic. The COVID-19 pandemic had minimal impact on consumers’ ability to access essential medicines. However, important challenges identified were poor availability of means of transportation, reduced income, and high cost of medicines, as well as fear of contracting the virus.

## INTRODUCTION

COVID-19 is a novel infectious disease caused by a newly discovered coronavirus strain SARS-CoV-2.^1^ It is an ongoing global pandemic first discovered in Wuhan, China, in 2019.^2,3^ The WHO declared the outbreak a public health emergency of international concern on January 30, 2020 and later characterized COVID-19 as a global pandemic on March 11, 2020.^4,5^ There are more than 11.3 million cases reported in more than 188 countries as of July 2020, with more than 531,000 deaths and 6.11 million recoveries.^6^

The emergence of COVID-19 has affected the economy globally by directly affecting production in key countries that are sole manufacturers of raw materials, intermediate products, and consumer goods, thereby creating supply chain and market disruption, and by its financial impact on firms and the financial markets.^7^

Essential medicines are those drugs that satisfy the priority healthcare needs of the population.^8^ As a result of the surge in the pandemic, which led to the inevitable lockdown of the economy across affected countries, there has been a noticeable decrease in production and exportation of raw materials as well as finished products (drugs) across different countries. These greatly affected the ease of access to these medicines by the consumers who need them either for treating acute ailments or for the management of chronic diseases. Most developing countries are in their early stages of pharmaceutical development; thus, they rely on importation of drugs, raw materials, and equipment from countries outside the region, notably India and China.^9^ Nigeria is highly dependent on other countries for its medicinal needs. About 70.0% of the medicines used in Nigeria and most other African countries are imported from China and India.^10^

Nigeria as a developing country is faced with the challenge of a weak healthcare system and inefficient drug supply chain management; this has been a major concern in the treatment of diseases and a major hindrance to the attainment of the universal health coverage and Sustainable Development Goal 3, which aim to ensure healthy lives and well-being for all. Access to medicine is an important component of good healthcare systems, and with uninterrupted access to medicine, improving the health outcome of the population is likely to be achieved.^11^

In Nigeria, the lockdown which was accompanied with the closure of borders and travel ban across states led to a significant drop in the quantity of essential medicines in the health facilities, making it difficult for consumers to get the medicines they need. The COVID-19 pandemic also caused an increase in the prices of medicines, hand sanitizers, face masks, personal protective equipment, and other medical equipment used for providing health care.^12^

This study therefore aims to examine the impact of the COVID-19 pandemic on access to essential medicines in Nigeria by end users.

## METHODS

### Study design and sampling technique.

This research was a cross-sectional survey of males and females aged 15 years and older residing in Nigeria at the time of data collection. We used a convenient sampling technique to recruit our study participants, who were required to fill the questionnaire within the 2-month time frame stipulated for the study.^13^ The inclusion criteria were being social media users and having access to Internet connection to fill out the questionnaire. We excluded health professionals who had higher chances of assessing medicines by virtue of their being essential workers.

### Study instrument and administration.

Questionnaire for the study was developed in several stages of drafts and reviews, guided by the literature and in consultation with specialists and experts in survey research. Pretest of the drafted questionnaire was undertaken among 15 respondents from the six geopolitical zones in Nigeria to examine face validity, readability, and comprehensibility. The final questionnaire has sections on the demographic and health characteristics of the respondents and a COVID-19 impact assessment.^14^ A link to the electronic questionnaire was shared to respondents across the 36 states of Nigeria using social media platforms, specifically WhatsApp and Facebook, because they form a suitable platform for disseminating information in Nigeria.^15^ We used the services of our social media networks to help distribute the questionnaires to their own networks so as to get more respondents. Data for the study were collected between May and June 2020. A total of 374 respondents completed and returned informed consent along with the questionnaire electronically.^16^

### Data analysis.

Data were coded and abstracted into the Microsoft Excel spreadsheet and thereafter loaded into the Statistical Package for the Social Sciences version 20 for final analysis. Simple descriptive analysis including frequencies and percentages was computed for demographic characteristics. We operationalized the respondents as those managing acute or chronic illnesses. Because this research was a national study, results were analyzed holistically, and thereafter relevant comparisons were made across the six geopolitical zones. First, the McNemar test was used to determine the differences between the sources of medication “before and during” the lockdown. It was also applied to determine the differences between difficulty of accessing essential medicines “before and during the lockdown.” Sources of medicines were dichotomized as “orthodox medicines” (essential medicines from hospitals, pharmacies, and patent medicine shops) and “alternative therapy” (essential medicines from herbal sources and no use of medicines) before McNemar’s test. Determination of ease of access to essential medicines among the different categories of illnesses (acute and chronic conditions). Difficulty of accessing medicine to worsening of chronic condition, increase in cost of medicine for acute and chronic conditions and across geopolitical zones, and, finally, impact of COVID-19 on the income of respondents was performed using chi-square test of independence and Fisher’s exact test, respectively. All statistical analyses were two-sided, with statistical significance defined at *P* < 0.05. Although the UN Sustainable Development Goal 3 envisions access to medicines by everyone, in this study, we operationalized minimum impact as 60.0% of the population being able to access medicines during the COVID-19 pandemic. Therefore, percentages less than 60.0% were considered as maximal impact on access to essential medicines, whereas percentages greater than that were considered as minimal impact.

## RESULTS

### Sociodemographic characteristics.

This research examined 374 respondents, with a majority (58.0%) of them aged between 16 and 30 years. More males (54.0%) participated in the study; more than two-third (75.5%) had tertiary education. A majority of the participants (34.2%) reside in the southeastern part of Nigeria; however, the North-east had the least respondent, which can arguably be attributed to poor access to the Internet caused by insurgency in the region. Although more were either in public or private employment, only 9.1% were unemployed at the time of this study (Table 1).

**Table 1:** Sociodemographic characteristics of the respondents

Variable	Frequency	Percent
Age-group (years)		
0–15	1	0.3
16–30	217	58.0
31–45	138	36.9
46–60	16	4.3
61+	2	0.5
Gender		
Male	202	54.0
Female	174	46.0
Educational qualification		
Primary	1	0.3
Secondary	6	1.6
Postsecondary	283	75.7
Masters/PhD	84	22.5
No formal education	0	0.0
Others	0	0.0
Geopolitical zone		
South-south	91	24.3
Southeast	128	34.2
Southwest	66	17.6
North-central	60	16.0
Northeast	9	2.4
Northwest	20	5.3
Employment		
Student	23	6.1
Self-employed	69	18.4
Employed in private/public sector	245	65.5
Unemployed	36	9.6
Other	1	0.3

*N* = 374.

### Health characteristics.

We found out that malaria and hypertension were the most prevalent acute and chronic disease conditions among respondents during the lockdown respectively (Table 2). McNemar’s test was conducted to compare the differences between source of essential medicines before and during COVID-19 lockdown. The proportion of respondents who sourced for orthodox medicines before COVID-19 lockdown (98.4%) was significantly (*P* < 0.001) higher than that of those who sourced for the same category of medicines during the COVID-19 lockdown (89.0%). However, 11.0% of the respondents sourced for alternative therapy compared with only 1.6% who used alternative therapy before the lockdown (Tables 3 and 4).

**Table 2:** Health characteristics of the respondents

Disease condition	Frequency	Percent
Acute conditions		
Malaria	107	41.5
Typhoid	29	11.2
Cold/flu	27	10.5
Urinary tract infection	10	3.9
Others	85	32.9
Chronic conditions		
Hypertension	82	42.7
Diabetes	54	28.1
Asthma	25	13.0
Arthritis	15	7.8
Others	33	17.2

**Table 3:** Source of essential medicines of respondents

Variable	Frequency	Percent	Frequency	Percent
Source of medicines	Before the pandemic		During the pandemic	
Hospital	69	18.4	33	8.8
Pharmacy/patent medicine shop	299	80.0	300	80.3
Herbal source	6	1.6	4	1.1
None	0	0.0	37	9.9

*N* = 374.

**Table 4:** Relationship between sources of essential medicines before and during the COVID-19 pandemic, and association between difficulty accessing medicine for chronic conditions “before and during” the COVID-19 lockdown

Sources of essential medicines before COVID-19 lockdown	Sources of essential medicines during COVID-19 lockdown		*P*-value
	Orthodox medicines, *N* (%)	Alternative therapy, *N* (%)	
Orthodox medicines	332 (88.8)	36 (9.6)	0.000
Alternative therapy	1 (0.3)	5 (1.4)	

Difficulty accessing essential medicine before the COVID-19 pandemic	Difficulty accessing essential medicine during the COVID-19 pandemic		*P*-value
	Yes, *N* (%)	No, *N* (%)	
Yes	8 (5.6)	7 (4.9)	0.000
No	42 (29.6)	85 (59.9)	

Orthodox medicine = hospitals, pharmacies, and patent medicine shops. Alternative therapy = herbal sources and no use of medicines. (*N* < 100% because of nonresponse in each case.)

We also found out that the pandemic had impact on the ease of essential medicine access for both acute and chronic conditions. For respondents living with chronic conditions, there was an increase in the proportion of those facing difficulties to essential medicine access, from 10.6% before the lockdown to 35.2% during the lockdown (Table 4). Of those who had difficulty assessing essential medicines for chronic conditions during the COVID-19 pandemic, 84.0% experienced a worsening of the chronic conditions, which was significant (Table 5). Overall, those who had acute illnesses during the lockdown had 72.0% essential medicines accessibility, whereas those with chronic conditions had 65.0% accessibility.

**Table 5:** Impact of the COVID-19 pandemic on access to essential medicines for acute condition, and association between difficulty accessing essential medicines and worsening of chronic condition

	Difficulty accessing required medicines during the pandemic		*P*-value
Variable	Yes, *N* (%)	No, *N* (%)	
Acute condition			
Yes	52 (28.0)	134 (72.0)	0.379
No	0 (0.0)	2 (100.0)	
Fisher’s exact value = 1.00; df = 1			
Worsening of chronic condition			
Yes	21 (84.0)	4 (16.0)	0.000
No	38 (27.9)	98 (72.1)	
Fisher’s exact value = 0.00; df = 1			

df = degree of freedom.

The COVID-19 pandemic has also been shown to affect the cost of essential medicines for both acute and chronic illnesses, as 75.0% of those with acute illnesses and 74.0% of those with chronic illnesses attested to an increase in the cost of medicines during the pandemic. Altogether, 77.3% of the respondents observed an increase in medicine costs (Supplemental Tables 1 and 2).

## DISCUSSION

This is the first study, to our knowledge, that investigates the impact of the COVID-19 pandemic on access to medicines for acute and chronic conditions among consumers in Nigeria. In this research, we operationalized minimum impact as 60.0% of the population being able to access medicines during the COVID-19 pandemic. Findings of this research demonstrated a minimum impact of the COVID-19 pandemic on the Nigerian population, whereby 72.0% and 65.0% of respondents managing acute and chronic illnesses, respectively, had access to essential medicines. This can be attributed to the fact that essential workers like healthcare providers did not face restrictions to movement or running their healthcare facilities during the lockdown.

An estimated 35.2% of the respondents managing chronic illnesses had difficulties accessing essential medicines during the COVID-19 pandemic lockdown compared with the only 10.6% who had difficulties accessing essential medicines before the lockdown. This is an issue of great concern considering the interaction between inadvertent nonadherence and health outcomes. We found that 84.0% of our respondents experienced deteriorating chronic health conditions in the light of difficulty in accessing essential medicines. A study demonstrated that comorbidities such as hypertension, diabetes, cardiovascular disease, and respiratory diseases could affect the outcome of COVID-19 infection.^17^ Arguably, the fear of contracting COVID-19 and the poor outcomes in background comorbidity deterred our respondents from seeking their medicines. We could not, however, ascertain if there were any healthcare provider–related influences on the inability to obtain required medicines during the COVID-19 pandemic lockdown. Therefore, it may be worthwhile to consider strategies such as community-based responses to supplement healthcare assess where people cannot get to healthcare facilities during pandemics. This is supported by the significant increased resort to the readily available “alternative therapy” during the pandemic as shown in McNemar’s test (Table 4).

Another study reported that a high percentage of healthcare workers agreed to participate in neighborhood response strategies, as this would reduce the influx of people to hospitals and allow already overstretched healthcare providers to focus their attention on more severe cases requiring their expertise.^18^ Furthermore, evidence suggests that better coordination of health care in addition to community-based care management approaches will provide suitable alternative to medicine when the healthcare system and, of course, access to health care are thwarted by a pandemic.^19^ Our study further underscored the importance of maintaining at least a 30-day supply of prescription and nonprescription medicines to reduce the frequency of health facility visit, especially for those living with chronic illnesses.^20^ However, this can compound the strain on incomes already caused by the pandemic. Our study showed that 73.9% of respondents living with chronic illness affirmed that their incomes were negatively affected by the pandemic, with a significant impact on income across the various categories of employment of the respondents. This can be directly linked to the abrupt halt in economic activities due to lockdown and restriction of movement.

The COVID-19 pandemic disrupted the national and international supply chain systems, including those of essential medicines and pharmaceutical products as a result of border closures, trade restrictions among nations, and transportation problems.^21^ Worse still, developing countries such as Nigeria are shrouded with complicated drug distribution chains with a consequent increase in drug cost as it gets to the end user.^22^ This problem is further compounded by the existence of a pandemic. Similar trends were observed in our study as 77.3% of the respondents reported an increase in medicine costs. The impact of the COVID-19 pandemic on drug security would be more significantly felt by developing countries as they rely heavily on imported drug products to meet their medication needs.^23^ This highlights the need for policy-makers in Nigeria to address challenges to large-scale and sustainable drug manufacturing, using the COVID-19 situation as a learning opportunity.

Because of the stay-at-home order imposed on nonessential workers, 73.9% of the study participants with acute and chronic conditions, respectively, confirmed that their sources of income were affected. Interestingly, only 10.0% of the Nigerian population has health insurance coverage.^24^ It could be argued that people who make out-of-pocket payments would most profoundly experience the impact of the COVID-19 pandemic on income generation and therefore their purchasing power.

**Figure 1.**
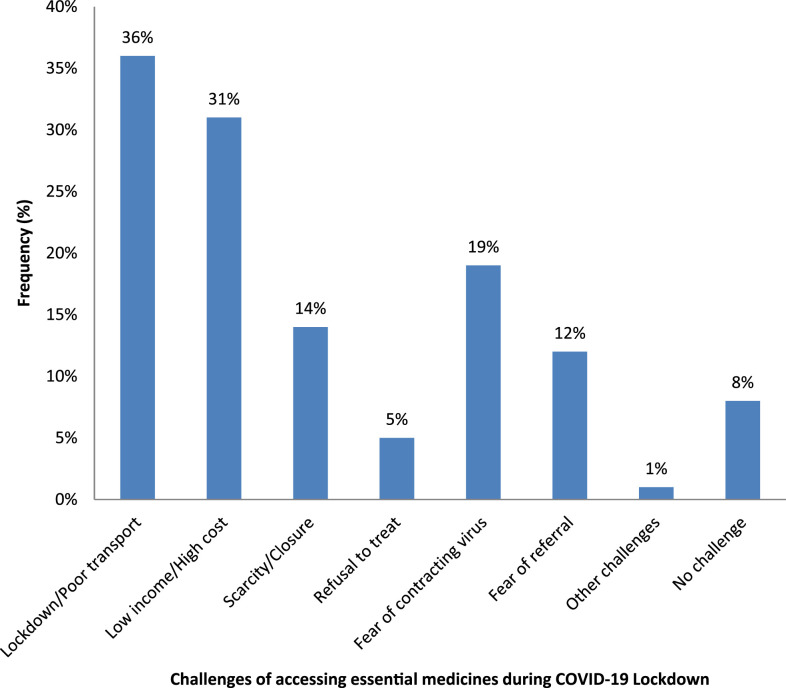
Frequency of challenges of accessing essential medicines during COVID-19 lockdown. This figure appears in color at www.ajtmh.org.

Our study identified poor means of transportation (36%), low income and high cost of medicines (31%), fear of exposure to the coronavirus during hospital visits (19%), scarcity of required medicines and closure of some healthcare facilities (14%), fear of referral to COVID-19 isolation centers (12%), and refusal of healthcare workers to attend to patients for fear of contracting the virus themselves (5%) as the major barriers to assessing essential medicines among respondents during the pandemic/lockdown (Figure 1).

Although this study identified a number of issues of public health significance, it is limited in the ability to control the inclusion and exclusion criteria, given the peculiarity of sampling methods and data collection strategies. In addition, we operationalized minimum impact as at least 60.0% of the population having access to essential medicine. This definition is peculiar to our setting where medicines are obtained from out-of-pocket expenditure in the light of poor price control policy, and this constituted an analytical constraint to the study. The study may have also faced social/response bias, a limitation common to self-administered questionnaire research. Nevertheless, the study underscored the imperative to strengthen supply chain systems and incorporate primary healthcare models in disease outbreak preparedness strategies.

## CONCLUSION

Our results highlighted that although the COVID-19 pandemic and the associated lockdown orders had minimal impact on consumers’ ability to access essential medicines, however, there were significant challenges and resultant effect associated with this impact. These include difficulties in assessing essential medicines for chronic illnesses with a resultant worsening of chronic conditions, increased medicine costs due to supply chain disruptions, decrease in income generation to make out-of-pocket payments for medicines, and a shift from where medicines were sourced. We, therefore, recommend that for a sustained access to medicines for other illnesses during pandemics, infectious disease outbreak preparedness strategies should incorporate primary healthcare services and other health system modalities to cater for non–pandemic-related conditions.

## Supplemental tables

Supplemental materials
