# Supplementary material for: Impact of the COVID-19 Pandemic on Consumers’ Access to Essential Medicines in Nigeria
Source: Am J Trop Med Hyg. 2020 Aug 18;103(4):1630–4. doi: 10.4269/ajtmh.20-0838 (PMC7543821; doi:10.4269/ajtmh.20-0838)
Supplement: Supplementary file 1 [file tpmd200838.SD1.pdf]

Supplemental Table 1: Association between increase in cost of medicines across the six geo-political zones of Nigeria and across the different categories of disease conditions.

Variable	Increase in cost of medicines during pandemic Yes N (%)	No N (%)	*P*-value
**Geo-political zones**			
South-east	100 (78.1)	28 (21.9)	0.650
South-south	70 (76.9)	21 (23.1)	
South-west	52 (78.8)	14 (21.2)	
North central	42 (70.0)	18 (30.0)	
North-east	8 (88.9)	1 (11.1)	
North-west	17 (85.0)	3 (15.0)	
Fisher's Exact Value = 0.727; df = 5			
**Disease condition**			
Acute conditions			
**YES N(%)**	142 (75.5)	46 (24.5)	0.419
**NO N(%)**	147 (79.0)	39 (21.0)	
Fisher's Exact Value = 0.460; df = 1			
Chronic conditions			
**YES N(%)**	142 (74.0)	50 (26.0)	0.116
**NO N(%)**	147 (80.8)	35 (19.2)	
Fisher's Exact Value = 0.139; df = 1			

Supplemental Table 2: Association between increased cost of medicines across the different categories of employment amongst respondents.

Variable	Impact of COVID-19 pandemic on income Yes N (%)	No N (%)	*P*-value
**Employment**			
Student	20 (87.0)	3 (13.0)	0.002
Self-employed	56 (81.2)	13 (18.8)	
Employed in private/public sector	154 (62.9)	91 (37.1)	
Unemployed	30 (83.3)	6 (16.7)	
Others	1 (100.0)	0 (0.0)	
Fisher's Exact Value = 0.001; df = 4			
**Diseases Condition**			
Acute condition	139 (73.9)	49 (26.0)	
Chronic condition	142 (73.9)	50 (26.0)	
